# Predictive factors analysis of cesarean scar pregnancy treated by local injection of Lauromacrogol combined with curettage

**DOI:** 10.1097/MD.0000000000032783

**Published:** 2023-01-27

**Authors:** Jianxia Huang, Pei He, Dingheng Li, Jianwei Zhou

**Affiliations:** a Gynecology Department, The Second Affiliated Hospital, Medical College of Zhejiang University, Hangzhou, Zhejiang, China; b Department of Obstetrics, Hangzhou Women’s Hospital (Hangzhou Maternity and Child Health Care Hospital), Hangzhou, Zhejiang, China; c Department of Gynecology, Hangzhou Women’s Hospital (Hangzhou Maternity and Child Health Care Hospital), Hangzhou, Zhejiang, China.

**Keywords:** cesarean scar pregnancy, curettage, Lauromacrogol, predictive value

## Abstract

To explore factors related to local injection of Lauromacrogol combined with curettage in the treatment of cesarean scar pregnancy. A total of 24 successful and 8 unsuccessful cases were included. The age, gravidity, parity, times of cesarean section, interval from the last cesarean section, preoperative human chorionic gonadotropin (HCG), HCG on the first day after operation, decreasing rate of HCG on the first day after operation, average diameter of gestational sac, and preoperative vaginal bleeding days were analyzed. There were no significant differences of age, gravidity, parity, previous cesarean section times between groups. The differences of preoperative HCG, HCG on the first day after operation, the decreasing rate of HCG, gestational sac diameter, preoperative vaginal bleeding days were statistically significant between groups. The interval from the last cesarean section and the decreasing rate of HCG were protective factors, while the mean diameter of gestational sac and period of vaginal bleeding before operation were risk factors for the success of the treatment. The mean diameter of gestational sac owned the best predictive value.

**Key points**

- Local injection of Lauromacrogol under ultrasound is an effective treatment for CSP with high successful rate, short hospitalization time, and quick recovery.
- There is still a certain failure rate of Lauromacrogol treatment in our study.
- The differences of preoperative HCG, HCG on the first day after operation, the decreasing rate of HCG on the first day after operation, gestational sac diameter, preoperative vaginal bleeding days were statistically significant between successful and unsuccessful groups treated by local injection of Lauromacrogol and curettage.
- Binary logistic regression model showed that the interval from last cesarean section, decrease rate of HCG on the first day after operation, mean diameter of gestational sac, and days of vaginal bleeding before operation were predictive factors.
- ROC curve showed that the average diameter of the gestational sac before operation had the best predictive value for the local treatment of cesarean scar pregnancy with Lauromacrogol.

## 1. Introduction

Cesarean scar pregnancy (CSP) is an iatrogenic result caused by previous cesarean section. It is very dangerous. CSP increased with the increasing rate of cesarean section. The incidence of CSP in women with a history of cesarean section ranged from 1/1000 to 1/2500.^[1]^ CSP increases the risk of bleeding, placenta abruption, placenta previa, and uterine rupture.^[1]^ If it was missed or handled improperly, CSP could lead to severe maternal complications at any gestational week. Timely diagnosis is needed, and the best therapy should be done in the first treatment. Ultrasound is a first line diagnostic tool. Although local minimally invasive treatment, systemic medication, laparoscopic, and hysteroscopic surgery have been used in clinic, the best treatment is still uncertain.^[2]^

Of all the treatments, local injection is the most minimally invasive, whether through bladder or vagina. Uterine artery embolization can reduce the fertility and pregnancy rate slightly, which may due to non-target embolism of some ovarian artery.^[3]^ Local injection of Lauromacrogol under ultrasound is an effective treatment for CSP with high successful rate, short hospitalization time, and quick recovery. Local injection of Lauromacrogol can block the blood vessels supplying gestational sac in cesarean scar and prevent bleeding. Lauromacrogol is a widely used hardener. Lauromacrogol can promote hardening in 2 ways. First, Lauromacrogol can cause venous fibrosis around the injection site, resulting in vascular compression, and hemostasis. Second, direct intravascular injection of Lauromacrogol can destroy endothelial cells in the affected vessels, promoting local thrombosis. The latter can lead to aseptic inflammation and tissue fibrosis, and the injured veins may develop into fibrous cords eventually.^[4]^ However, we found that there was still a certain failure rate of the local Lauromacrogol treatment, and it was very important for CSP patients to choose the best, and effective treatment at the first time.^[5]^ Therefore, the purpose of this study was to explore the predictors of local treatment with Lauromacrogol to improve the successful rate.

## 2. Materials and methods

### 2.1. Objects

A case-control study was performed. A total of 112 patients with CSP admitted to Hangzhou Obstetrics and Gynecology Hospital and Hangzhou Xiaoshan Hospital from January 2014 to December 2019 were collected. We screened the type II CSP patients. According to the principle of 3:1, We adopted the random number method, 24 successful and 8 unsuccessful cases were enrolled randomly. All patients signed informed consent, and this study was discussed and agreed by the hospital medical ethics committee (no. 2016-002-6).

### 2.2. Diagnostic and exclusive criteria

#### 2.2.1. Diagnostic criteria.

B-ultrasound showed that:

(1) The uterine cavity and cervical canal were clearly visible and did not contact with the capsule.
(2) The gestational sac was embedded in the uterus anterior isthmus, with or without fetal heart activity.
(3) Muscle layer defect (muscle layer shrinkage or loss) between bladder and sac.
(4) Abundant vascular images in scar area of cesarean section.^[4]^
CSP could be divided into 2 types, type I was to grow into uterine cavity, type II was to grow into serosa layer.^[6]^ All of enrolled patients were type II CSP and were treated with B-ultrasound guided local injection of Lauromacrogol around gestational sac followed by B-ultrasound guided curettage.

#### 2.2.2. Exclusive criteria.

Patients with severe heart, liver, brain, lung, and kidney diseases; active inflammatory disease; special medication history during pregnancy, complications such as hemorrhage, conversion to surgery, loss of follow-up, incomplete information, etc.

### 2.3. Methods

#### 2.3.1. Definition of successful treatment.

we treated the patients with curettage guided by B-ultrasound about 24 hours after local injection of Lauromacrogol. After the operation, serum human chorionic gonadotropin (HCG) decreased to normal range gradually without any other intervention.

#### 2.3.2. Injection of Lauromacrogol.

The bladder was emptied and 21-gauge PTC needle (Hakko, Tokyo, Japan) were used under the guidance of B-ultrasound. When the needle tip reached the gestational sac, 5 to 10 mL Lauromacrogol was injected slowly at multiple points until the blood flow signals around the gestational sac disappeared.

The decreasing rate of HCG on the first day after operation = (HCG before operation - HCG on the first day after operation)/ HCG before operation;

The average diameter of gestational sac = the sum of 3 diameters of gestational sac/ 3.

### 2.4. Statistical analysis

IBM-SPSS 25.0 (SPSS, Chicago, IL) was used for statistical analysis. One sample Kolmogorov Smirnov test was used to test the normality of data. The data of skew distribution were expressed as median and percentile [M (P25, P75)] and normal distribution data were expressed as mean ± standard deviation (¯x± s). The data of skew distribution were compared by Mann–Whitney *U* test. The data of normal distribution were compared by independent *t* test. Binary logistic regression was used to analyze the factors, and the Odds ratio, and 95% confidence interval (CI) of the related variables were calculated. cutoff and AUC were determined by receiver operating characteristic curve, and the predictive value of related indicators was evaluated. The optimal cutoff, AUC and Youden index were calculated. When *P* < .05, the difference was statistically significant.

## 3. Results

### 3.1. Comparison of basic indicators

The data of parity, cesarean section times, HCG before operation, HCG after operation, decreasing rate of HCG on the first day after operation, and days of vaginal bleeding before operation manifested as skew distribution. While the data of age, gravidity, interval of cesarean section, and mean diameter of gestational sac were normal distribution.

There were no significant differences of age, gravidity, parity, number of previous cesarean section between groups (all *P* > .05). The differences of preoperative HCG (*P* < .001), HCG on the first day after operation (*P* < .05), decreasing rate of HCG on the first day after operation (*P* = .007) average diameter of gestational sac (*P* = .007), preoperative vaginal bleeding days (*P* = .047) were statistically significant between groups. (Table 1).

**Table 1:** Comparison of basic data between successful and unsuccessful groups.

	Age (yr)	Gravidity	Parity	Times of caesarean section	Interval from last caesarean section (yr)	Preoperative HCG (IU/L)	HCG on the first day after operation (IU/L)	Decreasing rate of HCG	Mean diameter of gestational sac (cm)	Preoperative vaginal bleeding time (day)
Successful group	33.96 ± 3.97	3.38 ± 1.17	1.0 (1.0–2.0)	1.0 (1.0–1.0)	6.54 ± 3.68	27,697 (14,053.75–48,609.5)	12,020 (5765.5–15483)	60.43% (53.93%–72.47%)	1.34 ± 0.65	0.5 (0−2.0)
Unsuccessful group	31.88 ± 3.00	3.13 ± 1.13	1.0 (1.0–2.0)	1.0 (1.0–2.0)	3.38 ± 2.26	123,420 (81,872.25–184,421.75)	75,653 (39,916–99,690.5)	43.23% (31.64%–54.67%)	2.83 ± 1.14	6.0 (0.25−19.5)
*t* or *Z*	1.354	0.527	−0.67	−0.928	2.278	−4.091	−4.178	−2.698	−3.508	−1.985
*P*	.186	.602	.503	.353	.03	< .001	< .001	.007	.007	.047

HCG = human chorionic gonadotropin.

### 3.2. Results of binary logistic regression analysis

Binary logistic regression model was used to analyze the related factors. The results showed that the Odds ratio of interval of cesarean section, decrease rate of HCG on the first day after operation, mean diameter of gestational sac, and days of vaginal bleeding before operation were 0.709 (95% CI: 0.504–0.998), 0 (95% CI: 0–0.221), 14.619 (95% CI: 1.53–139.655), and 1.214 (95% CI:1.018–1.448), respectively. While HCG before operation and HCG on the first day after operation had no predictive value of local Lauromacrogol treatment. (Table 2)

**Table 2:** Binary logistic regression analysis of the related indicators and local Lauromacrogol treatment combined with curettage.

Indicators	β	SE	Wald	df	*P* value	OR	95% CI for OR	
							Lower	Upper
Interval from last caesarean section	−0.344	0.174	3.886	1	.049	0.709	0.504	0.998
HCG before operation	0	0	0.964	1	.326	1	1	1.001
HCG on the first day after operation	0.004	0.402	0	1	.992	1.004	0.457	2.205
Decreasing rate of HCG	−7.918	3.271	5.861	1	.015	0	0	0.221
Gestational sac	2.682	1.151	5.427	1	.02	14.619	1.53	139.655
Vaginal bleeding time	0.194	0.09	4.637	1	.031	1.214	1.018	1.448

CI = confidence interval, HCG = human chorionic gonadotropin, OR = odds ratio.

### 3.3. Predictive value of factors related to local Lauromacrogol treatment combined with curettage

**Figure 1.**
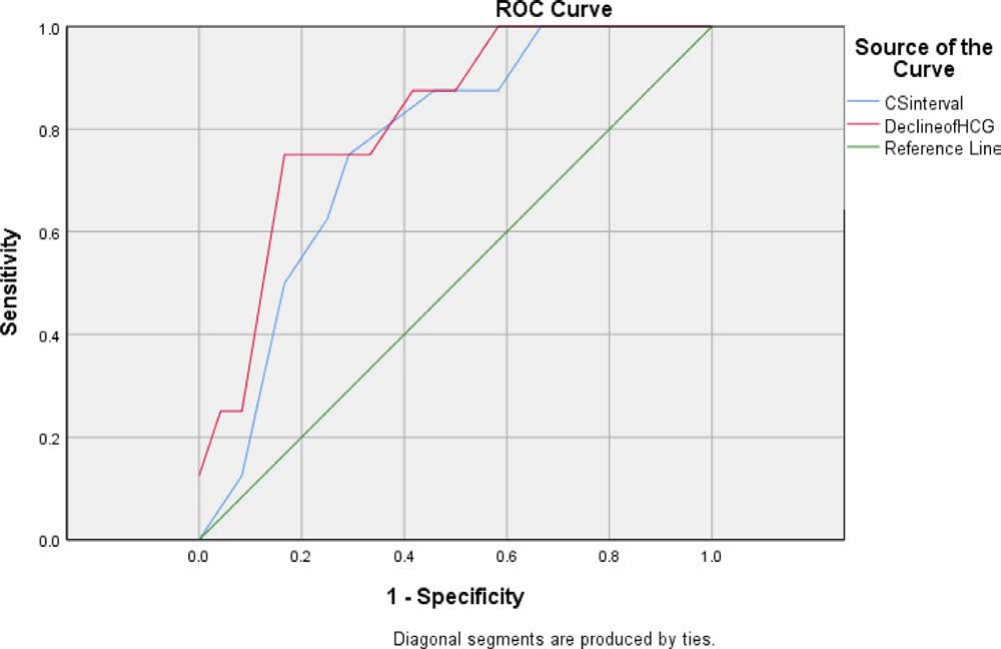
ROC curves of interval from the last cesarean section, decreasing rate of HCG on the first day after operation to the success of local Lauromacrogol combined with curettage treatment prediction. CSinterval: interval from the last cesarean section; decline of HCG: decreasing rate of HCG on the first day after operation. The AUC of interval from the last cesarean section, decreasing rate of HCG on the first day after operation were 0.763, 0.823, respectively. HCG = human chorionic gonadotropin, ROC = receiver operating characteristic.

**Figure 2.**
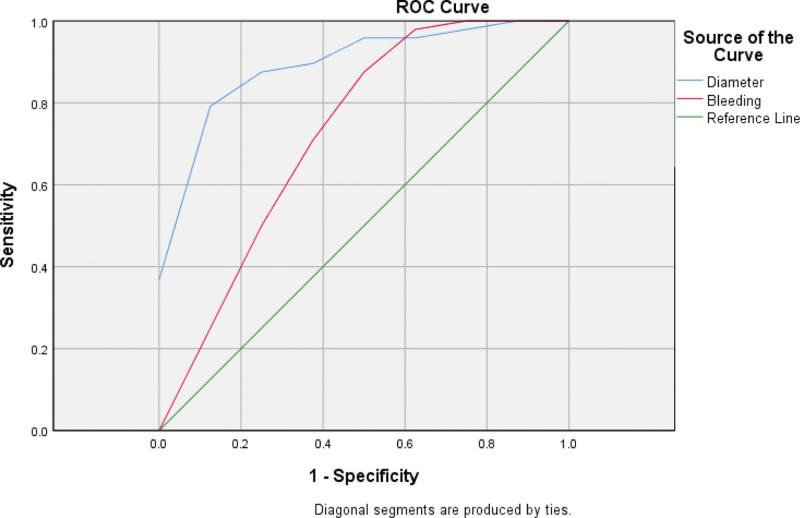
ROC curves of mean diameter of gestational sac, days of vaginal bleeding before operation to local Lauromacrogol combined with curettage treatment prediction. Diameter: mean diameter of gestational sac; Bleeding: days of vaginal bleeding before operation. The AUC of mean diameter of gestational sac, days of vaginal bleeding before operation were 0.917, 0.727, respectively. ROC = receiver operating characteristic.

The receiver operating characteristic curves of interval of cesarean section, decreasing rate of HCG on the first day after operation, diameter of gestational sac, and days of vaginal bleeding before operation were made. The results showed that the AUC, cutoff, sensitivity, specificity, and Youden index of interval of cesarean section to the treatment of local Lauromacrogol combined with curettage were 0.763, 4.5, 0.708, 0.75, 0.458, respectively. While decreasing rate of HCG on the first day after operation were 0.823, 50.35%, 0.875, 0.75, 0.625; diameter of gestational sac were 0.917, 1.78, 1,0.75, 0.75; days of vaginal bleeding before operation were 0.727, 9, 0.5, 0.958, 0.458, respectively (Table 3, Figs. 1–2).

**Table 3:** Predictive value of the related factors for the success of local Lauromacrogol injection combined with curettage treatment.

Indicators	AUC	95% CI	Cut-off	Unit	Sensitivity	Specificity	Yoden index	*P* value
Interval from the last caesarean section	0.763	0.589–0.937	4.5	yr	0.708	0.75	0.458	.028
Decreasing rate of HCG	0.823	0.665–0.981	50.35%	-	0.875	0.75	0.625	.007
Gestational sac	0.917	0.821–1.0	1.7833	cm	1	0.75	0.75	.02
Vaginal bleeding	0.727	0.494–0.959	9	d	0.5	0.958	0.458	.058

CI = confidence intervtal, HCG = human chorionic gonadotropin.

## 4. Discussion

CSP refers to the implantation of gestational sac on the scar of previous cesarean section, accounting for 6.1% of all ectopic pregnancy after cesarean section.^[7]^ However, the specific cause of CSP is still unclear.^[7]^ Uterine artery embolization combined with hysteroscopic resection, dilatation and curettage is considered to be a safe and effective treatment for CSP. The main purpose of uterine artery embolization is to block bilateral uterine arteries, reduce vaginal bleeding risk during curettage, and accelerate the decomposition of gestational sac. However, these treatments have a high incidence of complications, such as postoperative pain, fever, ovarian dysfunction, intrauterine adhesions, etc.^[8,9]^ Paolo Casadio reported that local methotrexate injection followed by hysteroscopic removal did not reduce fertility rate.^[10]^ But methotrexate is a chemotherapeutic drug, it may have some toxic side effects compared with Lauromacrogol. In fact, ultrasound-guided Lauromacrogol injection is easy to operate. It does not need large equipment. The patients can be free of radiation and the postoperative complications and treatment costs are lower. But not all patients are suitable for local injection of Lauromacrogol. There was still a certain failure rate of Lauromacrogol treatment in our study. However, the higher successful rate of Lauromacrogol treatment reported by Ze Ying Chai et al^[4]^ may be related to the short pregnancy peroid and low preoperative HCG level of the enrolled patients in their study. For CSP patients, timely diagnosis and proper treatment measures are particularly important.

Our results showed that there were significant differences of interval of cesarean section, HCG before operation, diameter of gestational sac between groups. This was basically consistent with the results of previous studies on CSP. Guangquan Liu et al^[11]^ showed that gestational sac diameter, preoperative HCG, and gestational weeks were significantly positively correlated with intraoperative bleeding. HCG on the first day after operation, the decreasing rate of HCG on the first day after operation were different between groups as well. As Li Qiuyang et al^[12]^ showed, HCG declined slowly, and needed a longer recovery time in patients with large gestational sac and high HCG levels.

Furthermore, binary logistic regression analysis showed that the interval of cesarean section, decreasing rate of HCG on the first day after operation, diameter of gestational sac and days of vaginal bleeding before operation were related to the success of Lauromacrogol treatment. The interval of cesarean section and the HCG declining rate on the first day after operation were protective factors of Lauromacrogol treatment, the gestational sac, and the days of vaginal bleeding before operation, were risk factors of Lauromacrogol treatment. This was consisted with the existing studies. Xianyi Zhou et al^[7]^ showed that interval less than 5 years from the last cesarean section was a high risk factor for CSP. The gestational sac larger than 5 cm and HCG decreasing rate less than 66.42% were the risk factors of persistent CSP.^[13]^ The longer the time of vaginal bleeding before operation, the severer the fibrosis around the gestational sac, which enhanced the tolerance of the gestational sac to ischemia. Therefore, the longer the preoperative vaginal bleeding time, the lower the success rate of Lauromacrogol local treatment. However, the preoperative and postoperative HCG levels had little predictive value for the local treatment of Lauromacrogol. Considering that the basic HCG levels are large and different from individual to individual.

Further analysis showed that the average diameter of gestational sac before operation was the best index to predict the success of Lauromacrogol treatment, which may provide direction for future treatment. This was consistent with previous studies. Multivariate analysis by Ma Y et al^[14]^ showed that only the largest diameter of the gestational sac was significant in the retrospective equation, which could predict intraoperative bleeding.

However, the current study had several limitations. Firstly, this was a retrospective study. Secondly, the number of patients in each group was limited. Larger prospective studies are needed in the future.

## 5. Conclusions

In conclusion, this study suggested that the average diameter of the gestational sac before operation was the best predictive value for local treatment of CSP with Lauromacrogol.

## Acknowledgments

We thank all members of the research group.

## Author contributions

**Data curation:** Dingheng Li.

**Methodology:** Jianxia Huang, Pei He.

**Project administration:** Pei He, Jianwei Zhou.

**Supervision:** Jianwei Zhou, Jianxia Huang.

**Writing – original draft:** Jianxia Huang.
